# An Efficient Modern Strategy to Screen Drug Candidates Targeting RdRp of SARS-CoV-2 With Potentially High Selectivity and Specificity

**DOI:** 10.3389/fchem.2022.933102

**Published:** 2022-07-12

**Authors:** Haiping Zhang, Xiaohua Gong, Yun Peng, Konda Mani Saravanan, Hengwei Bian, John Z. H. Zhang, Yanjie Wei, Yi Pan, Yang Yang

**Affiliations:** ^1^ Shenzhen Institute of Synthetic Biology, Shenzhen Institutes of Advanced Technology, Chinese Academy of Sciences, Shenzhen, China; ^2^ Shenzhen Key Laboratory of Pathogen and Immunity, National Clinical Research Center for Infectious Disease, State Key Discipline of Infectious Disease, Shenzhen Third People’s Hospital, Second Hospital Affiliated to Southern University of Science and Technology, Shenzhen, China; ^3^ Department of Biotechnology, Bharath Institute of Higher Education and Research, Chennai, , India; ^4^ Shanghai Engineering Research Center of Molecular Therapeutics and New Drug Development, Shanghai Key Laboratory of Green Chemistry and Chemical Process, School of Chemistry and Molecular Engineering, East China Normal University, Shanghai, China; ^5^ Center for High Performance Computing, Joint Engineering Research Center for Health Big Data Intelligent Analysis Technology, Shenzhen Institutes of Advanced Technology, Chinese Academy of Sciences, Shenzhen, China

**Keywords:** antiviral agent, RdRp, compound specificity estimation, virtual screening, tubeimoside III

## Abstract

Desired drug candidates should have both a high potential binding chance and high specificity. Recently, many drug screening strategies have been developed to screen compounds with high possible binding chances or high binding affinity. However, there is still no good solution to detect whether those selected compounds possess high specificity. Here, we developed a reverse DFCNN (Dense Fully Connected Neural Network) and a reverse docking protocol to check a given compound’s ability to bind diversified targets and estimate its specificity with homemade formulas. We used the RNA-dependent RNA polymerase (RdRp) target as a proof-of-concept example to identify drug candidates with high selectivity and high specificity. We first used a previously developed hybrid screening method to find drug candidates from an 8888-size compound database. The hybrid screening method takes advantage of the deep learning-based method, traditional molecular docking, molecular dynamics simulation, and binding free energy calculated by metadynamics, which should be powerful in selecting high binding affinity candidates. Also, we integrated the reverse DFCNN and reversed docking against a diversified 102 proteins to the pipeline for assessing the specificity of those selected candidates, and finally got compounds that have both predicted selectivity and specificity. Among the eight selected candidates, Platycodin D and Tubeimoside III were confirmed to effectively inhibit SARS-CoV-2 replication *in vitro* with EC_50_ values of 619.5 and 265.5 nM, respectively. Our study discovered that Tubeimoside III could inhibit SARS-CoV-2 replication potently for the first time. Furthermore, the underlying mechanisms of Platycodin D and Tubeimoside III inhibiting SARS-CoV-2 are highly possible by blocking the RdRp cavity according to our screening procedure. In addition, the careful analysis predicted common critical residues involved in the binding with active inhibitors Platycodin D and Tubeimoside III, Azithromycin, and Pralatrexate, which hopefully promote the development of non-covalent binding inhibitors against RdRp.

## Introduction

### RdRp Is an Important Therapeutic Target of SARS-CoV-2

The global pandemic caused by SARS-CoV-2 has continued for more than 2 years and caused a huge threat to public health and the global economy. Variants carrying numerous mutations in the spike protein of SARS-CoV-2, which result in higher transmissibility and immune evasion of the current vaccines, and therapeutic monoclonal antibodies emerged during the transmission (Tao et al., 2021; Flemming, 2022). Unlike S protein, the RdRp of SARS-CoV-2 is highly conserved and plays a critical role in the virus replications, making it a potential therapeutic target to combat SARS-COV-2 (V’kovski et al., 2021). There are already remdesivir (Yin et al., 2020) and molnupiravir (Lee et al., 2021) drug that target RdRp, and our previous work also identify Pralatrexate and Azithromycin which may target RdRp and shows strongly block virus replication in cells (Zhang et al., 2020b).

### Drug Virtual Screening Has Been Accelerated by Deep Learning

Drug virtual screening has a very long history, with many related techniques have been developed, such as docking, Quantitative Structure-Activity Relationship (QSAR), pharmacophore, and structure-based ligand similarity. As emerging of deep learning algorithms and the experimental protein-ligand interaction dataset accumulates, deep learning-based protein-ligand interaction will greatly promote virtual drug screening. Currently, there are graphic-based protein-ligand interaction models that use a graphic representation of ligand or protein and can grasp the spatial and physical-chemical feature concisely; also, the graphic convolution network was used as training architecture (Torng and Altman, 2019). Also, a 4D CNN-based protein-ligand interaction model directly uses the X, Y, and Z coordinates of a protein-ligand complex, plus the extra atom feature as input (Stepniewska-Dziubinska et al., 2018). There are many other types of deep learning-based protein-ligand interaction prediction models, such as DeepDTAF (Wang et al., 2021), DeepBindRG (Zhang et al., 2019b), and DeepAffinity (Karimi et al., 2019). We also developed several protein-ligand interaction models, and some of them have successfully been applied to virtual screening applications for targets such as RdRp (Zhang et al., 2020b), 3C proteases (Zhang et al., 2020a), and TIPE2 (Zhang et al., 2021). For those protein-ligand binding prediction models, some predict binding affinity, whereas some are binary predictions of the binding possibility. Since the number of unbinding compounds usually dominates over the binding compounds in the virtual screening scenarios, binary prediction models that have considered the unbinding data during the training are more suitable for virtual screening.

### Current Virtual Screening Problem

A more effective integrated virtual screening strategy should be proposed. Deep learning-based protein-ligand prediction, molecular docking, and molecular dynamics simulation can be used for virtual screening. Each has different pros and cons in terms of efficacy and accuracy. However, the virtual screening database can be very large since the potential druglike compound size can be large as 10^60^. Hence, maintaining the efficiency and accuracy of the virtual screening is essential. The virtual drug screening can also be used for *de novo* compounds by integrating the deep learning-based *de novo* compounds generative model (Gupta et al., 2018). Many more advanced deep learning generative models based on RNN architecture have been gradually developed (Moret et al., 2021; Creanza et al., 2022). Among them, the beam search algorithm proposed by Michael Moret, et al., has great potential to generate more reasonable novel compounds (Moret et al., 2021). Beam search sampling overcomes the need for external scoring methods and extends the applicability of machine learning-driven molecular design. And they have tried to better adapt them to VS procedures and take into account some of VS limitations. Also, a new strategy called sampling with substitutions (SWS) usually generates molecules structurally similar to bioactive compounds or with given desired properties (Creanza et al., 2022). It generates molecules structurally similar to bioactive compounds or with given desired properties. It partly solves the problem that exploring a new chemical space could make the following synthesis difficult and expensive. Those newly developed methods also have the potential to preserve drug-likeness and synthetic accessibility of the generated *de novo* compounds. They can help discover *de novo* compounds with better binding affinity or other desired properties, such as better specificity or drug-likeness.

Furthermore, the current virtual screening often has not fully considered compounds’ specificity. Without specificity, there is a risk of potential side effects and cause the drug failure in a later stage. Currently, most virtual screening procedures don’t consider specificity, which can lead to obtaining pain compounds that have side effects and fail to pass the clinic test. Some researchers proposed a local-specific test strategy (Yan and Wang, 2012). However, estimating the given compound’s binding affinity with many diversified targets is necessary to test the relative global specificity. Hence, assessing the specificity of the compound by computational methods efficiently requires high-speed protein-ligand prediction tools (such as DFCNN) in the first stage. If a compound can bind a target that has significantly high affinity compared to other proteins, then such binding can be defined as high specificity and high affinity. Contrary, if a compound can bind to many proteins tightly, then it has a risk of low specificity.

### Our Current Work

This work identifies candidates from three TargetMol datasets (Targetmol-Approved_Drug_Library, Targetmol-Natural_Compound_Library, and Targetmol-Bioactive_Compound_Library) that can potentially bind to a larger range of RdRp cavity. The hybrid pipeline has effectively integrated deep learning-based method (DFCNN, DeepBindBC), docking (Autodock Vina), specificity checking (through reverse DFCNN, reverse docking against 102 representative proteins from DUD.E), and force field-based screening (pocket MD simulation and metadynamics simulation). In a step-by-step manner, the high binding affinity compounds are accumulated while the specificity is also considered. The proposed pipeline enables the screening of large compounds dataset with high efficiency while increasing the chance to obtain high affinity and high specificity compounds. With the proposed screening strategy, we obtained eight candidates of SARS-CoV-2 for experimental validation. Finally, we identified that Platycodin D and Tubeimoside III could inhibit SARS-CoV-2 replication *in vitro* with IC50 values of 619.5 and 265.5 nM, respectively. Unlike our previous work, we used a large pocket definition and considered the specificity during the screening; the obtained active compounds should be more specific and more diversified.

## Methods

### The Target and Compounds Database

Our previous work obtained the RdRp-ligand complex (Zhang et al., 2020b). In that work, the RdRp sequence and its modeled structure were obtained from https://zhanglab.ccmb.med.umich.edu/C-I-TASSER/2019-nCov/. The RdRp-ligand model was constructed by I-TASSER (Zhang, 2008), whose ligand was taken from the template protein (PDB ID: 3BR9) (Zhou et al., 2008) by COFACTOR algorithm (Roy et al., 2012) within I-TASSER using structure comparison and protein-protein networks. Unlike previous work, we extract the amino acids within 1.2 nm of the ligand as the binding pocket instead of 1 nm since the RdRp cavity is very large. The three TargetMol datasets (Targetmol-Approved_Drug_Library, Targetmol-Natural_Compound_Library, and Targetmol-Bioactive_Compound_Library) were used as virtual screening libraries. These datasets have diversified compounds including active compounds, natural compounds, and approved compounds. Moreover, most of the compounds can be easily pursued, we deposited the compound structures in GitHub (https://github.com/haiping1010/targetmol_datasets) for the convenience of other users.

### Molecular Vector-Based and Structure-Based Drug Screening

Similar to our previous work (Zhang et al., 2020b; Zhang et al., 2021), we have used a hybrid screening strategy that first uses molecular vector-based as initial drug screening and structure-based screening for further selection. The molecular vector-based relies on our previously developed DFCNN (Zhang et al., 2019a). The parameter setting for the DFCNN is the same as our previous work, which is drug repurposing against RdRp of SARS-CoV-2 (Zhang et al., 2020b), except that this time we used a large pocket definition, which defines residues with 1.5 nm from the predicted ligand as a pocket. The structure-based screening depends on DeepBindBC and autodock vina (Trott and Olson, 2010). The DeepBindBC prediction relies on the interface information of docked protein-ligand interface. By incorporating the cross-docking (docking proteins and ligands from different experimental complexes) conformation as negative training data, DeepBindBC can distinguish non-binders. The pocket is determined by the location of the ligand in the template protein. We set the cavity volume space with 3.5, 3.5, and 3.5 nm in *x*, *y*, and *z* dimensions from the pocket mass center. Other settings are the same as our previous work (Zhang et al., 2020b, 2021).

### The Reverse DFCNN Prediction and Reverse Docking to Access the Specificity of the Selected Compounds

To fast estimate the specificity for large amounts of compounds, we first use the DFCNN to make the reverse prediction against 102 proteins from DUD.E. We have defined a function to estimate the DFCNN-based specificity. The formula is used as follows:

$$\mathrm{specificity}=\log_{10}\left(103/\left(N_{c1}+1\right)\right)$$

where $N_{c1}$ is the counted number of compounds that have a DFCNN score larger than 0.99 during the reverse DFCNN prediction with 102 protein targets.

However, the DFCNN has not considered the spatial information; hence we carried a reverse docking by Autodock Vina for further relative specificity. The relative specificity is calculated by following formulas.

$$\mathrm{Relative}\;\mathrm{specificity}=\log_{10}\left(103/\left(N_{c2}+1\right)\right)$$

where $N_{c2}$ is the counted number of compounds that have an autodock vina score smaller than the mean autodock vina score of 102 protein-compound complexes during the reverse autodock vina docking with 102 protein targets. Notably, the smaller autodock vina score indicates better binding affinity.

### Force Field-Based Screening

Further drug screening was carried out by pocket molecular dynamics (MD) simulations and metadynamics similar to our previous work (Zhang et al., 2020b; Zhang et al., 2021), except that we used a large pocket definition (residues with 1.5 nm from the docked compounds was kept as pocket). This study selected 26 compound binding complexes, which were predicted candidates by previous deep learning screening and specificity checking, for MD simulation. Metadynamics simulations can estimate binding free energy surfaces to explore whether protein-ligand prefers to bind in solution. Metadynamics simulations facilitate the sampling of the free energy landscape and a specific collective variable of interest by adding a history-dependent biasing potential (Laio and Gervasio, 2008; Saleh et al., 2017). The detailed procedure of pocket molecular dynamics and metadynamics simulation was described as follows.

The initial protein-compound complexes were from the top score conformation Autodock Vina docking, the ligand was edited by pymol software (DeLano, 2002) to make it in the correct protonation state at pH 7. In this study, we selected 26 compound binding complexes that have a high DFCNN score (larger or equal to 0.99), low Vina score (low than −9 kcal/mol), large DeepBindBC score (larger or equal than 0.99), and especial high reverse DFCNN based specificity (larger or equal than 1.3) and reverse Autodock Vina based relative specificity (larger or equal than 0.6). We also refined a pocket molecular dynamics simulation (pocket MD, Supplementary Figure 11B) to facilitate the simulation process by only keeping the binding pocket region for simulation. Binding free energy calculation can be estimated by metadynamics simulations to explore whether protein-ligand will bind in solution. Metadynamics relies on the addition of a bias potential to sample the free energy landscape along with a specific collective variable of interest (Laio and Gervasio, 2008; Saleh et al., 2017). Note that the binding free energy calculations from Metadynamics may only be suitable for detecting the general trend of binding in virtual screening.

The pocket MD is same as the classical MD simulation, except that we only using the pocket region to reduce system size for simulation (Zhang et al., 2020b), which is inspired by a previous dynamic undocking (DUck) method (Ruiz-Carmona et al., 2017). An in-house script was used to extract the pocket region of the protein (here, we used 1.2 nm within the binding ligand), the N terminal and C terminal ends were capped with the ACE and NHE terminals, respectively. We applied position restrains to the ACE and NHE terminals to maintain the relative conformation of the pocket. MD simulation was carried out by Gromacs with AMBER-99SB force field (Hornak and Simmerling, 2003; Hess et al., 2008). The topology of the ligand and the partial charges of ligand was generated by ACPYPE (Sousa Da Silva and Vranken, 2012), which relies on Antechamber (Wang et al., 2006). First, we created a dodecahedron box and put the target-ligand complex at the center. A minimum distance from the protein to box edge was set to 1 nm. We filled the dodecahedron box with TIP3P water molecules (Jorgensen et al., 1983), the counter ions were added to neutralize the total charge using the Gromacs program tool (Van Der Spoel et al., 2005). The long-range electrostatic interactions under the periodic boundary conditions was calculated with Particle Mesh Ewald approach (Darden et al., 1993). A cutoff of 14 Å was used for van der Waals non-bonded interactions. Covalent bonds involving hydrogen atoms were constrained by applying the LINCS algorithm (Hess et al., 1997).

We performed the energy minimization steps with a step-size of 0.001 ns, 100 ps simulation with isothermal-isovolumetric ensemble (NVT), and 10 ns simulation with isothermal-isobaric ensemble (NPT) for water equilibrium. After that, a 100 ns NPT production run (step size 2 fs) was carried out. The Parrinello-Rahman barostat and the modified Berendsen thermostat were used for simulation with a fixed temperature of 308 K and a pressure of 1 atm. RMSD and hydrogen bond number of the trajectory were calculated using Gromacs tools.

The simulation was continued using the metadynamics approach for exploring the free energy landscape. The interface coordination number of atoms of protein-ligand complex was used as a collective variable (CV). The protein-ligand interface coordination numbers correlate with the numbers of atom contact, and larger coordination number usually indicates that the protein-ligand is in a binding state.

The coordination number *C* is defined as follows by Plumed:

$$C=\sum_{i\in A}\sum_{j\in B}S_{ij}$$ (1)

and

$$S_{ij}=\frac{1-{\left(\frac{r_{ij}-d_{0}}{r_{0}}\right)}^{n}}{1-{\left(\frac{r_{ij}-d_{0}}{r_{0}}\right)}^{m}}$$ (2)

In the simulation, *n* was 6, *m* was 12, $d_{0}$ was 0 nm and $r_{0}$ was 0.5 nm. $d_{0}$ is a parameter of the switching function. $r_{ij}$ is the distance between atom *i* and atom *j*. The degrees of contacts between two groups of atoms can be estimated by the above function (1) (Tribello et al., 2014). Metadynamics simulation for each protein-ligand system was performed for 100 ns (except protein-Azithromycin, which was extended to 300 ns in order to reach the 0 Coordination Number and achieve convergences). During the metadynamics simulation, Gaussian values were deposited every 1 ps with a height of 0.3 kJ/mol. The widths of the Gaussians were 5 for the coordination number. The free energy landscapes of the metadynamics simulations along the CV were generated by the Plumed program and plotted using Gnuplot (Williams et al., 2012).

### Tools Used in the Analysis

The USCF Chimera, VMD, ICM-browserPro, and Discovery Studio Visualizer 2019 were used to generate the structure and visualize the 2D protein-ligand interactions (Humphrey et al., 1996; Pettersen et al., 2004; BIOVIA, 2005; icm_browser_pro, 2020).

### Viral Stock Titration by 50% Tissue Culture Infective Dose

TCID_50_ was measured as previously reported (Yang et al., 2022). Briefly, Vero cells in 96-well plates were grown to 80% confluence and infected with 10-fold serial dilutions of the stock SARS-CoV-2 (hCoV-19/China/SZTH-025/2021, GISAID No. EPI_ISL_11799984) for 1 h at 37°C. Then the inoculum was removed, and cells were overlaid with fresh DMEM plus 2% FBS. Plates were assessed for the lowest dilution in which 50% of the wells exhibited cytopathic effects on the fifth-day post-infection (d.p.i). The values of TCID_50_ were calculated according to the Reed-Muench method (Reed and Muench, 1938).

### Evaluating the Antiviral Activities of the Candidate Drugs in Vero Cells

The antiviral activities of the drugs were evaluated as previously reported with some modifications (Zhang et al., 2020b). Vero cells were seeded at 4 × 10^4^ cells per well in 24-well plates and allowed to adhere for 24 h, the virus (MOI ≈ 0.02) and different doses of the indicated drugs were added to allow infection for 1 h at 37°C. Then viral inoculum was removed, and cells were washed 2 times with PBS. Then the cells were further cultured with fresh DMEM with 2% FBS and the indicated concentrations of drugs. At 48 h post-infection (h.p.i), the cell supernatant was collected, and viral RNAs were extracted using the QIAamp RNA Viral Kit (Qiagen, Heiden, Germany) for further quantification analysis using quantitative reverse transcription-polymerase chain reaction (qRT-PCR) was performed using a commercial kit (Mabsky Biotech Co., Ltd.). All the experiments involving infectious SARS-CoV-2 were handled in BSL-3 facilities at the Shenzhen Third People’s Hospital. The dose-response curves were plotted from viral RNA copies versus the drug concentrations using GraphPad Prism 8 software.

### Cytotoxicity Testing Assay

The cytotoxicity of Platycodin D and Tubeimoside III on Vero cells was evaluated by the Cell Counting Kit-8 (CCK-8, Beyotime Biotech) according to the manufacturer’s protocols. In brief, Vero cells grown in 96-well plates were treated with various concentrations of compounds or mock-treated for 24 h, followed by 4 h incubation in the media supplemented with 10% CCK-8. Viable cells were counted according to the absorbance at 450 nm.

## Results

**FIGURE 1:**
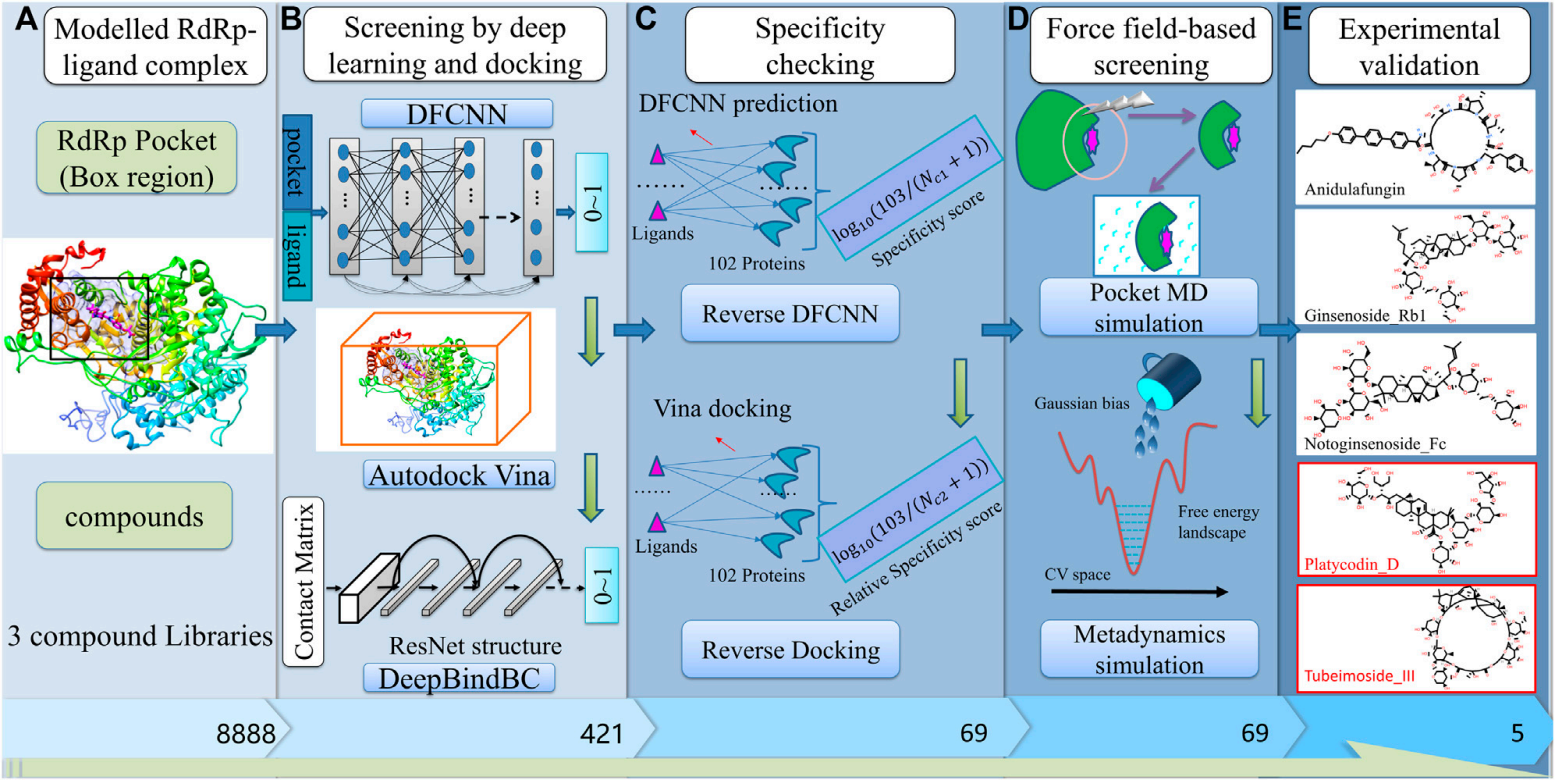
The pipeline of the virtual screening procedure against RdRp. **(A)** build RdRp-ligand model, and prepare three compounds libraries (Targetmol-Approved_Drug_Library, Targetmol-Natural_Compound_Library, and Targetmol-Bioactive_Compound_Library); **(B)** Deep learning and docking-based screening by DFCNN, Autodock Vina and DeepBindBC; **(C)** Specificity checking by reverse DFCNN and reverse docking against 102 diversified targets from DUD.E dataset; **(D)** Fine screening by pocket MD simulation and Metadyanmics simulation; **(E)** Experimental validation of final selected candidates.

The overall workflow is illustrated in Figure 1, which is composed of several stages, including pocket determination and compound libraries preparation. (Figure 1A); preliminary screening by deep learning and docking (Figure 1B); specificity checking by Reverse DFCNN and Reverse Docking; Fine screening by pocket MD and metadynamics simulation; most important, we experimentally validate the candidates in the final step. Notably, among eight compounds that were finally obtained from our hybrid screening strategy, two compounds (Platycodin D and Tubeimoside III) are active in inhibiting the SARS virus with IC50 values of 619.5 nM 265.5 nM, respectively. Overall, the hybrid virtual screening procedure is designed for compounds with a high chance of binding and high specificity against a given target.

The obtained candidates from preliminary screening are shown in Table 1. Since the pipeline methods have different advantages and are often complementary to each other, for instance, the DFCNN achieves extremely high efficiency by only considering the physical-chemical feature of pocket and ligand through vector representation. At the same time, the Autodock Vina and DeepBindBC have also considered spatial information. Hence, we selected those compounds based on their high DFCNN score (larger or equal to 0.99), low Vina score (low than −9 kcal/mol), large DeepBindBC score (larger or equal than 0.99), and especial high reverse DFCNN based specificity (larger or equal than 1.3) and reverse Autodock Vina based relative specificity (larger or equal than 0.6). The specificity check aims to eliminate those compounds that may bind to multiple off-targets, hence guaranteeing the relative safety of the candidates in the late stage of drug development.

**TABLE 1:** The result list with corresponding prediction scores after gradually screening by DFCNN, Autodock Vina, DeepBindBC, DFCNN specificity checking, and Autodock Vina relative specificity checking.

Name	DeepBindBC	Vina (kcal/mol)	DFCNN	Sp1*	C1*	Sp2*	C2*
Tubeimoside_I	1.000	−12.8	0.996	1.708	1	2.013	0
Tubeimoside_III	1.000	−14.4	0.991	1.708	1	2.013	0
Anidulafungin	0.999	−10.8	0.990	1.531	2	1.708	1
Ciwujianoside-B	1.000	−12.3	0.994	1.531	2	1.230	5
Platycodin_D	1.000	−12.2	0.997	1.398	3	1.146	6
Chikusetsusaponin_IV	1.000	−11.5	0.998	1.398	3	1.041	8
Polygalasaponin_V	1.000	−12.4	0.998	1.531	2	1.041	8
Officinalisinin_I	1.000	−11.3	0.993	1.708	1	1.000	9
Ginsenoside_Rb1	1.000	−10.6	0.990	1.708	1	0.954	10
Protodioscin	1.000	−11.4	0.995	1.398	3	0.954	10
Dipsacoside_B	1.000	−11.6	0.997	1.531	2	0.903	11
Deapi-platycodin_D	1.000	−11.6	0.996	1.398	3	0.845	13
Ginsenoside_Ro	1.000	−11.7	0.997	1.531	2	0.845	12
Pulsatilla_saponin_D	1.000	−12	0.998	1.398	3	0.845	12
anemarsaponin_B	1.000	−10.9	0.993	1.531	2	0.778	16
Esculentoside_H	1.000	−10.8	0.997	1.531	2	0.778	14
Timosaponin_BII	1.000	−10.7	0.993	1.708	1	0.778	14
Astragaloside_IV	1.000	−10.9	0.997	1.398	3	0.699	19
Eleutheroside_E	1.000	−9.2	0.992	1.531	2	0.699	19
Ginsenoside_Rd	1.000	−10.3	0.993	1.398	3	0.699	18
Asiaticoside	1.000	−11.1	0.998	1.398	3	0.602	22
Ginsenoside_Rc	1.000	−10.4	0.992	1.708	1	0.602	20
Hederacoside_D	1.000	−11.2	0.997	1.531	2	0.602	22
Notoginsenoside_Fc	1.000	−10.1	0.990	1.708	1	0.602	22
Rebaudioside_A	1.000	−10.9	0.995	1.398	3	0.602	24
Syringaresinol-di-O-glucoside	1.000	−9.2	0.992	1.531	2	0.602	24

Sp1: DFCNN specificity.

C1: the number of DFCNN scores in the predictions against 102 protein targets larger or equal than 0.99.

Sp2: Vina relative specificity.

C2: the number of vina scores in the docking against 102 protein targets is larger or equal to the known compound-RdRp vina score.

### The Final Candidates From MD Simulation and Metadynamics Simulation

The 26 candidates from the preliminary screening were considered for 100 ns pocket MD simulations. Among them, we focused on the eight candidates that showed stability in the last 50 ns simulation, which fulfilled conditions of average RMSD < 0.5 nm, standard deviation < 0.1 nm or RMSD < 0.6 nm, and standard deviation < 0.5 nm. The RMSD values of these eight pocket-compound complexes are shown in Supplementary Figure S1A. Furthermore, we find many hydrogen bonds formed between the pocket and ligand of these eight candidates, which show in Supplementary Figure S1B. Among the eight compounds, the Anidulafungin is an approved drug, and recent reports have shown that Anidulafungin has anti-SARS-nCov-2 active with IC50 of 4.64 μM (Jeon et al., 2020), and other researcher’s docking results support that it may bind to NSP12 (RdRp) (Dey et al., 2021). The other seven compounds are mostly from TCM herbal ingredients; for instance, the Ginsenoside_Rb1 and Ginsenoside_Rd can be isolated from ginseng (Lu et al., 2009), and Notoginsenoside_Fc is an ingredient from the leaves of Panax notoginseng (Yang et al., 1983).

**FIGURE 2:**
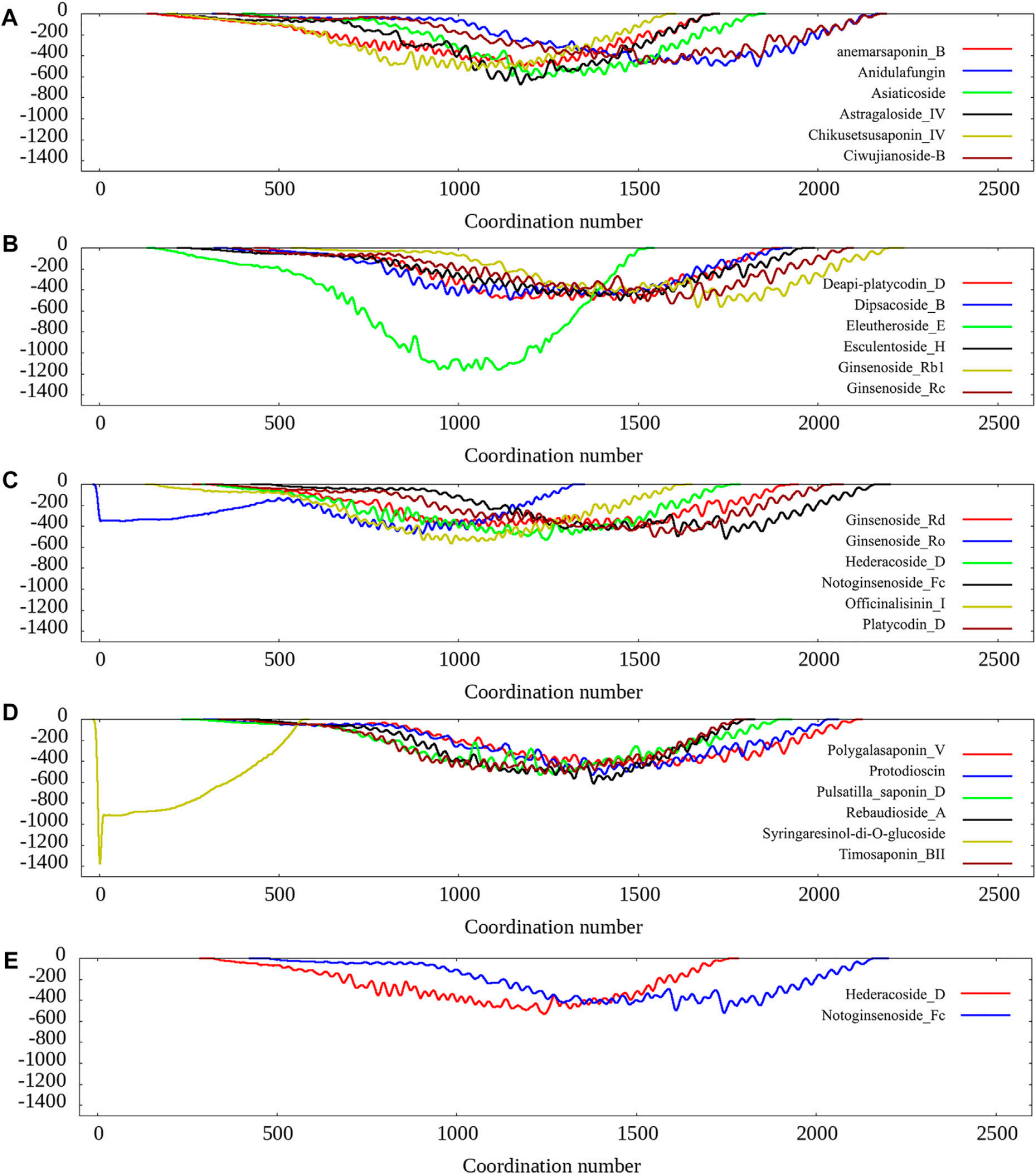
The calculated free energy landscape from the 100 ns metadynamics simulations.

We also carried 100 ns metadynamics for the 26 pocket-ligand complexes to further evaluate the ligand-pocket binding free energy landscape. The results show that most of the protein-ligands prefer to bind except for a few cases such as Syringaresinol-di-O-glucoside, Ginsenoside_Ro, as shown in Figure 2. Since the eight compounds selected in the previous stage all show preferring to bind according to the free energy landscape, we choose them as the final candidates for experimental validation.

**FIGURE 3:**
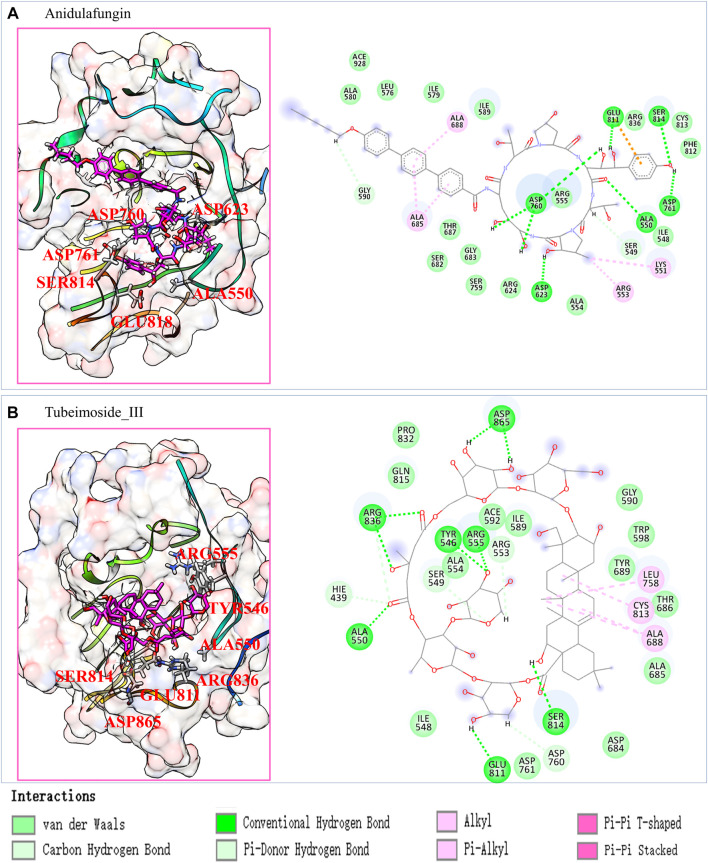
The predicted Anidulafungin and Tubeimoside_III interact with the RdRp pocket. **(A)** the last frame of RdRp pocket-Anidulafungin complexes from 100 ns MD simulation and its 2D interaction plot. **(B)** the last frame of RdRp pocket-Tubeimoside_III complexes from 100 ns MD simulation, and its 2D interaction plot.

To explore the detailed interaction between pocket and ligand, we have analyzed their interaction in atomic details. We noticed that Anidulafungin and Tubeimoside_III are compounds that contain a large cyclic substructure, which is consistent with our previous work that Azithromycin is an active inhibitor against SARS-nCov-2 that contain cyclic structure (Zhang et al., 2020b), and highly possible through binding to RdRp through our screening prediction. The interaction patterns of these two candidates from the last frame of MD simulation are shown in Figure 3.

Other three candidates with ligand average RMSD < 0.5 nm and standard deviation < 0.1 nm during the last 50 ns MD simulation are Ginsenoside_Rb1, Notoginsenoside_Fc, and Platycodin_D. Their interaction with the RdRp pocket from the last frame conformation of the 100 ns MD simulation is shown in Supplementary Figure S2. We observed that these three candidates contain glucan substructures and form many hydrogen bonds with the RdRp. Compared with our previous screening work, it is the first time we discovered that glucan substructure might be able to play an essential role in interacting with RdRp large pocket.

With different selecting criteria, we may obtain some other potential drug candidates as well. Here, we tried the criteria of ligand average RMSD < 0.6 nm and standard deviation < 0.5 nm in the last 50 ns MD simulation and obtained the other three candidates, which are shown in Supplementary Figure S3. Similarly, they also contain a glucan substructure, and all form many hydrogen bonds with the RdRp pocket.

### Platycodin_D and Tubeimoside_III Inhibit the Replication of SARS-CoV-2 *In Vitro*

**FIGURE 4:**
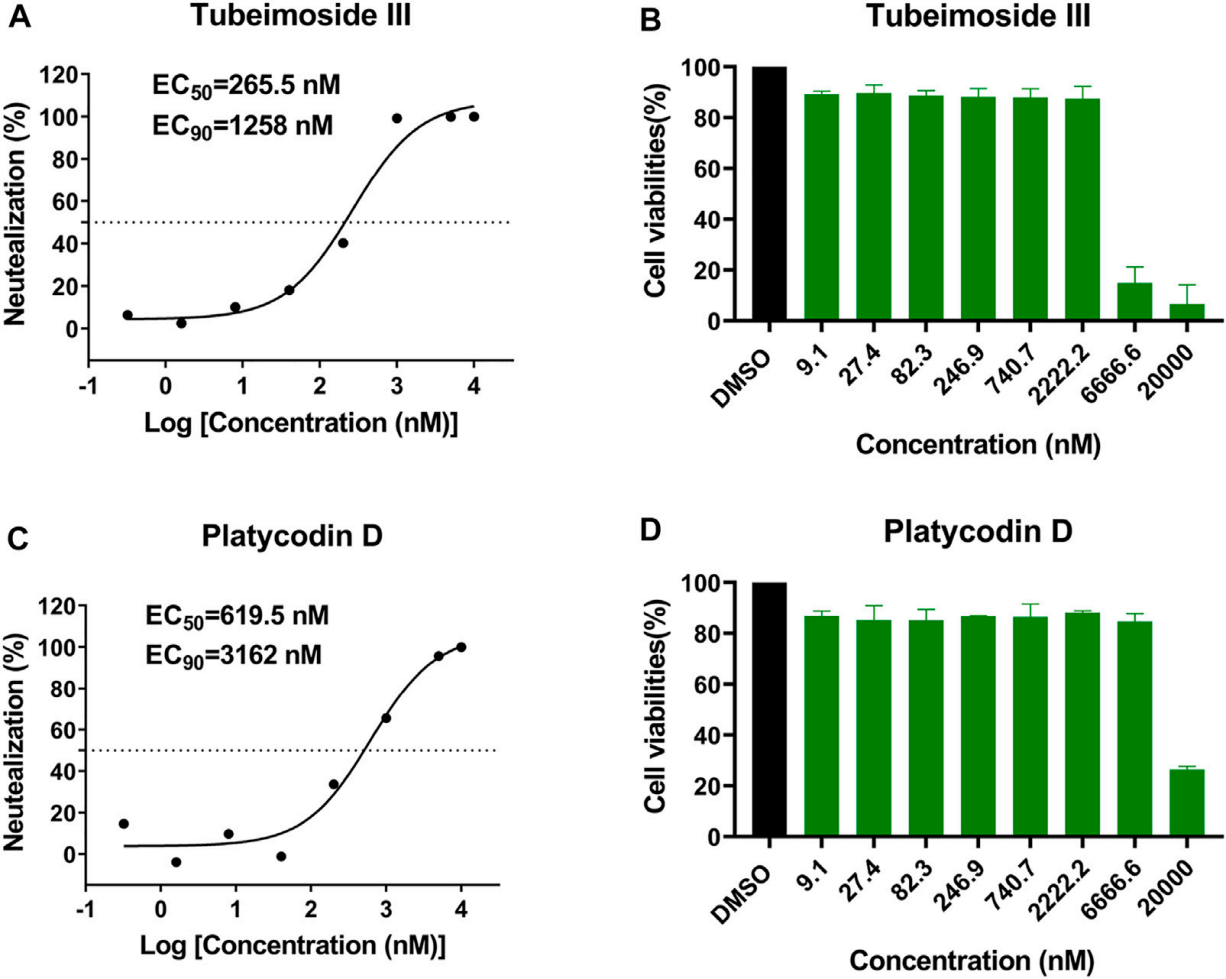
The antiviral activities of Platycodin_D and Tubeimoside_III against SARS-CoV-2 and cytotoxicity in Vero cell. **(A,C)** Vero cells were infected with SARS-CoV-2 at an MOI of 0.02 in the presence of the indicated concentrations of the tested drugs for 48 h, and the viral yield in the cell supernatant was then quantified by qRT-PCR. The dose-response curves were plotted from viral RNA copies versus the drug concentrations using GraphPad Prism 8 software. **(B,D)** Vero cells grown in 96-well plates were treated with various concentrations of compounds or mock-treated for 24 h, followed by 4 h incubation in the media supplemented with 10% CCK-8. Viable cells were counted according to the absorbance at 450 nm.

To further confirm the efficiency of virtual screening, we tested the antiviral activity of the eight candidate drugs *in vitro*, including Anidulafungin, Ginsenoside_Rb1, Notoginsenoside_Fc, Platycodin_D, Tubeimoside_III, Ginsenoside_Rd, Polygalasaponin_V, and Rebaudioside_A. Similar to our previous study (Zhang et al., 2020b), Vero cells were infected with SARS-CoV-2 at an MOI of 0.02 in the presence of varying concentrations of the tested drugs, and the inhibition rates were evaluated by quantification of viral copy numbers in the cell supernatant via qRT-PCR (Figure 4). The results showed that Platycodin_D and Tubeimoside_III could efficiently inhibit the replication of SARS-CoV-2, with half-maximal effective concentration (EC_50_) values of 619.5 and 265.5 nM, and EC_90_ of 3,162 and 1,258 nM, respectively. Although both Platycodin_D and Tubeimoside_III showed high cytotoxicity in the CCK8 assay (Figure 4), the two drugs showed little cytotoxicity even at the concentrations of EC_90_.

## Discussion

Compound specificity is extremely important for drug development. In contrast, our first-stage drug screening has seriously ignored this or lacks an effective way to assess this. Due to low specificity would not cause the failure of direct later experimental validation of protein-ligand binding affinity and cell activity. However, the ultimate goal of drug screening is to find compounds that can pass the preclinical stage and have good potential to pass the clinical stage and finally benefit patients through providing the on-market available drugs. Experimentally evaluating the compound specificity is expensive. This work demonstrates that with the increased efficiency and accuracy of protein-ligand prediction models, it is possible to evaluate the specificity by reverse compound-target assessment in the early stage of drug development. Dual or multi-targeting inhibitors are important for some complicated diseases (Ramsay et al., 2018), such as cancer, some research pointed out that next-generation anticancer agents may be dual or multi-targeting inhibitors (Raghavendra et al., 2018). A similar strategy as the reverse target searching in specificity check may provide new insight for screening and designing dual or multi-target compounds. Obtaining compounds for one given target is already a challenging task, to obtain dual or multi-target compounds would require a much more efficient new method and strategy, our proposed DFCNN and autodock vina-based evaluation multi-ligand VS multi-target binding relationship may provide some clues for future development of dual or multi-target compounds.

**FIGURE 5:**
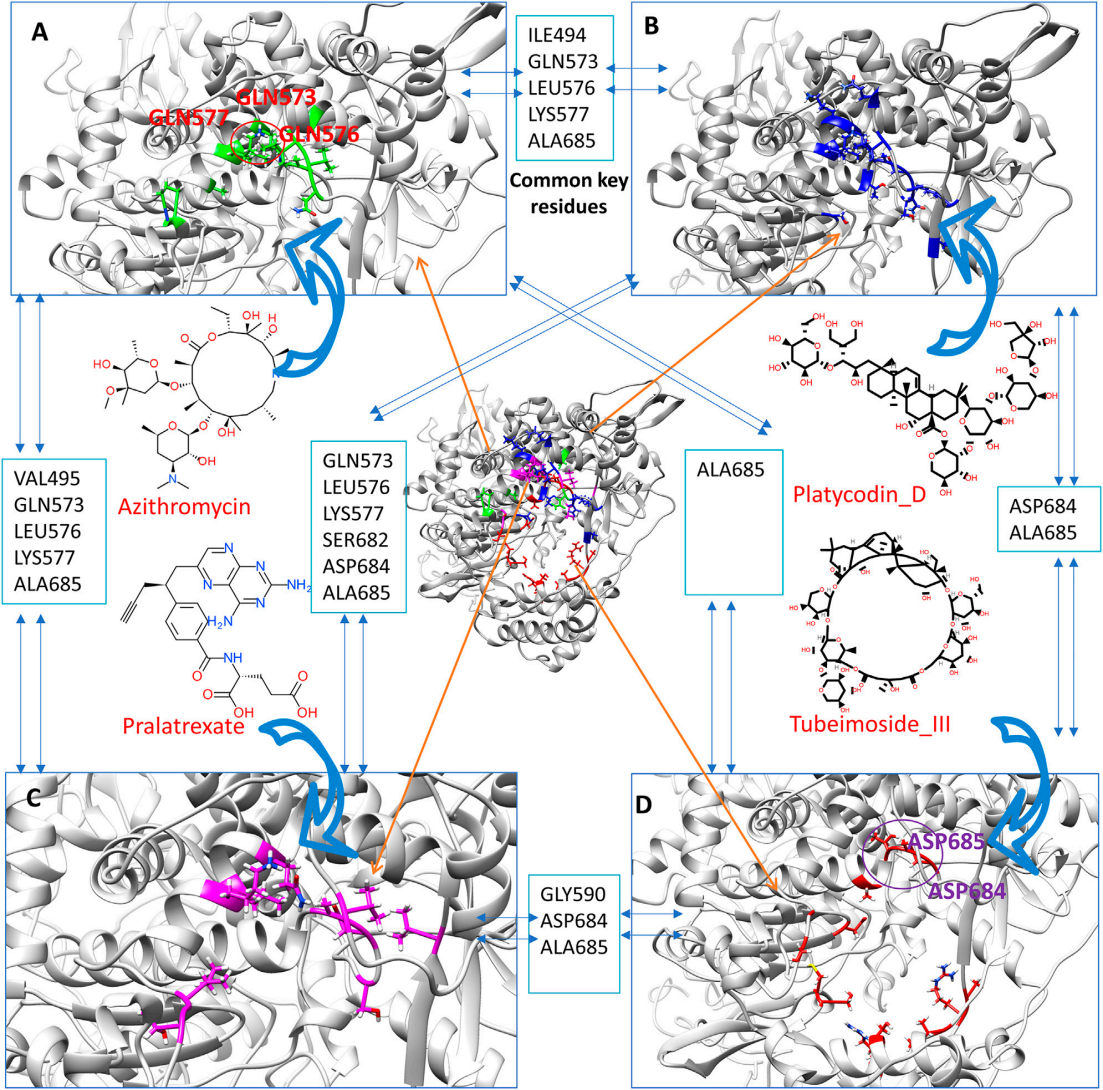
The close contact residues (within 0.5 nm) of the four active inhibitors from their last frame of MD simulation. The close contact residues of Azithromycin (**A**, residue with green), Platycodin_D (**B**, residues with green), Pralatrexate (**C**, residues with magenta), and Tubeimoside_III (**D**, residues with magenta). The common key residues between each of these compounds’ close contact residues are also listed.

To assess whether any specific active binding sites in the large cavity of RdRp are more likely to bind active inhibitors, we compare the binding site of Platycodin_D and Tubeimoside_III and our previous obtained Azithromycin and Pralatrexate from their last frame of MD simulation. Figure 5 shows that many common key residues (close contact residues within 0.5 nm of the binding compound) are among the four active compounds. For instance, there are three common key residues (GLN573, LEU576, LYS577) among Azithromycin, Platycodin_D, and Pralatrexate, indicating the region occupied by these three residues is critical for active ligand binding. Unlike the Azithromycin, Platycodin_D, and Pralatrexate, which bind mainly in a small cavity region, the Tubeimoside_III has interacted with more key residues around the whole cavity, which forms a cycle. Tubeimoside_III also has some common key residues with the other three active compounds. Altogether, the four compounds can potentially bind to the large cavity of RdRp, which is a site for DNA binding. This analysis strongly supports our hypothesis that ligands that can strongly bind to the cavity of RdRp can block the DNA premier entry into the cavity, hence potentially stopping the viral replications. Notably, a recent paper underlined that phospholipidosis is a shared mechanism underlying the antiviral activity of many repurposed drugs (Tummino et al., 2021). In other words, the antiviral activity found for many compounds could be an artifact. Since we first obtain candidates through screening over RdRp, it should have a much low chance to obtain compounds that inhibit viral through phospholipidosis compared to those directly screening through cell experiment. We carefully examine the four found active compounds with those compounds with phospholipidosis mentioned in the paper. Also, we have checked the literature report about whether each compound involves phospholipidosis. We find that azithromycin can induce phospholipidosis according to the reports (Liu et al., 2015). Hence, azithromycin’s anti-SARS-CoV-2 effect in the cell may not only through binding with the RdRp. For the other three compounds (Platycodin_D, Tubeimoside_III, and Pralatrexate), no literature report show they can induce phospholipidosis. Furthermore, their physical-chemical feature is not like those compounds that can induce phospholipidosis, which indicates compounds selected by our method more tends to exert inhibiting effect by binding with the protein target.

To assess the general features of those low specificity compounds that have high potential affinity but low DFCNN specificity (Supplementary Figure S4A) or autodock vina-based relative specificity (Supplementary Figure S4B) and select those compounds to have Vina relative specificity larger than 1 from Table 1. For better comparison, we also list cases that have high calculated specificity cases in Supplementary Figure S4C. We observed that high specificity compounds are usually composed of more unique groups. However, it is not easy to distinguish high specificity from abundant compounds by direct visual observation; hence, specificity calculation is necessary to determine the compound’s specificity quickly. In this work, two specificity scores were used, the first one using the DFCNN, which mainly considers binding through the pocket and ligand physicochemical property, and the second one which use autodock vina docking, which can provide structure insight about specificity. In terms of selectivity, we have used MD simulation and metadynamics simulation, which provide atomic details and dynamics information about structural interaction between compound and target.

The RdRp (nsp12) is highly conserved among the subtypes, and it only has one mutation (P323L) in Omicron (Ilmjärv et al., 2021). To explore whether the mutation can influence the large cavity, we have marked P323 residue with yellow in Supplementary Figure S5 from the nsp12-nsp7-nsp8 complex with PDB ID 7ED5 (Shannon et al., 2022). It should be noted that it is far away from the Template-primer RNA binding cavity. This strongly supports our assumption that active compounds binding to RdRp of original SARS-CoV-2 should also become active against Omicron. It is also interesting to note that the ligand AT9 is covalent binding to the Template-primer RNA to stop its elongation. At the same time, our hit compounds aim to prevent the Template-primer RNA from entering the cavity.

Since we are exploring compounds inhibitory RdRp, the most possible off-target would be some other polymerases that similar to RdRp in humans. We have checked the 102 targets and found there is one poly (ADP-ribose) polymerase (PARP) (PDB ID: 3l3m). It would be much more helpful if it contains more other polymerases, especially DNA polymerase in this study, since the compounds that prefer binding with RdRp may also prefer binding with human DNA polymerase, and lead to unwanted side effects. We suspect this is the reason why the selected compounds still have slight cell toxicity. And strongly suggest that in some specific cases, the user should add more relevant protein to the target pool, in order to obtain compounds with high specificity. For instance, in the future, it should use similar method to check whether the compounds will bind to human DNA polymer when designing RdRp inhibitors, in order to reduce the undesired side effects.

The compound ADME is very important. We have calculated the ADME property of the final selected inhibitors by the online SwissADME (http://www.swissadme.ch/). Most calculations suggest that our selected compounds have too larger a molecular weight. Hence, it is very important to analyze structural features essential to simplify those inhibitors while keeping their activities. Also, the topological polar surface area (TPSA) of a molecule is also too large. Take Platycodin_D as an example, removing some of its glycan groups may improve its MADE property. But we should note that some on-market drugs also break the Lipinski rule of five. Breaking one or more of Lipinski’s rules, does not mean a drug candidate cannot be effective. Moreover, compounds beyond the rule of five are often better suited for investigation when targeting large and flat binding sites (Egbert et al., 2019). Interestingly. the RdRp pocket is actually very large.

## Conclusion

In the present work, we identify highly potential RdRp inhibitors that may bind at a different site of a large RdRp cavity by screening more compounds. Also, we selected more specific candidates by integrating the specificity checking procedures into the screening pipeline. Moreover, we have discovered that Platycodin_D and Tubeimoside_III have a strong inhibiting effect against SARS-CoV-2 with IC50 values of 619.5 and 265.5 nM, respectively. Notably, our methods strongly indicate similarities to Azithromycin and Pralatrexate. Their inhibitory effect is highly likely through competitive prevention of the RNA template-primer RNA entering into its RdRp binding cavity. In addition, we have explored the detailed binding sites of these RdRp nonbonded inhibitors (Azithromycin, Platycodin_D, Pralatrexate, and Tubeimoside_III) and illustrated their binding mechanism by comparing some of the common key residues. Altogether, we again show the workability and power of the hybrid screening strategy, bringing in specificity checking methods, which can be applied to a wide range of drug screening applications, and illustrated the binding mechanism of RdRp nonbonded inhibitors. Also, the screening pipeline will hopefully become a generalized drug screening pipeline against many other therapeutic protein targets in the future.

## Data Availability

The original contributions presented in the study are included in the article/Supplementary Material, and further inquiries can be directed to the corresponding authors.
