# Supplementary material for: An Efficient Modern Strategy to Screen Drug Candidates Targeting RdRp of SARS-CoV-2 With Potentially High Selectivity and Specificity
Source: Front Chem. 2022 Jul 12;10:933102. doi: 10.3389/fchem.2022.933102 (PMC9315156; doi:10.3389/fchem.2022.933102)
Supplement: Supplementary file 1 [file DataSheet1.docx]

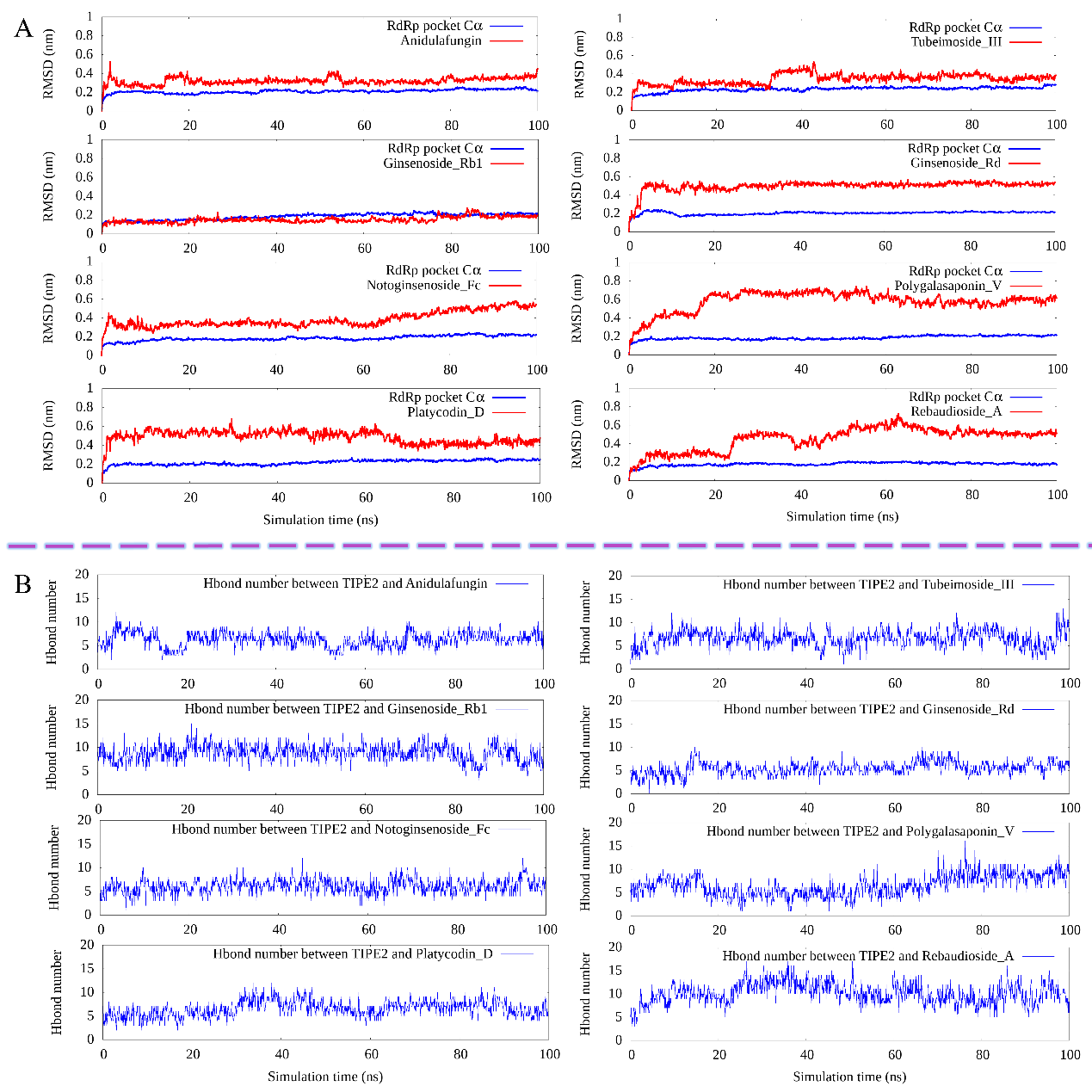


**Figure S1.** The RMSD and number of hydrogen bond plots for 8 pocket-ligand complexes that have fulfilled conditions of ligand average RMSD < 0.5 nm, standard deviation< 0.1 nm or ligand average RMSD < 0.6 nm, and standard deviation < 0.5 nm in the last 50ns MD simulation. Panel A shows the RMSD value of protein CA and ligand during the MD simulation. Panel B shows the number of hydrogen bonds formed during the MD simulation.


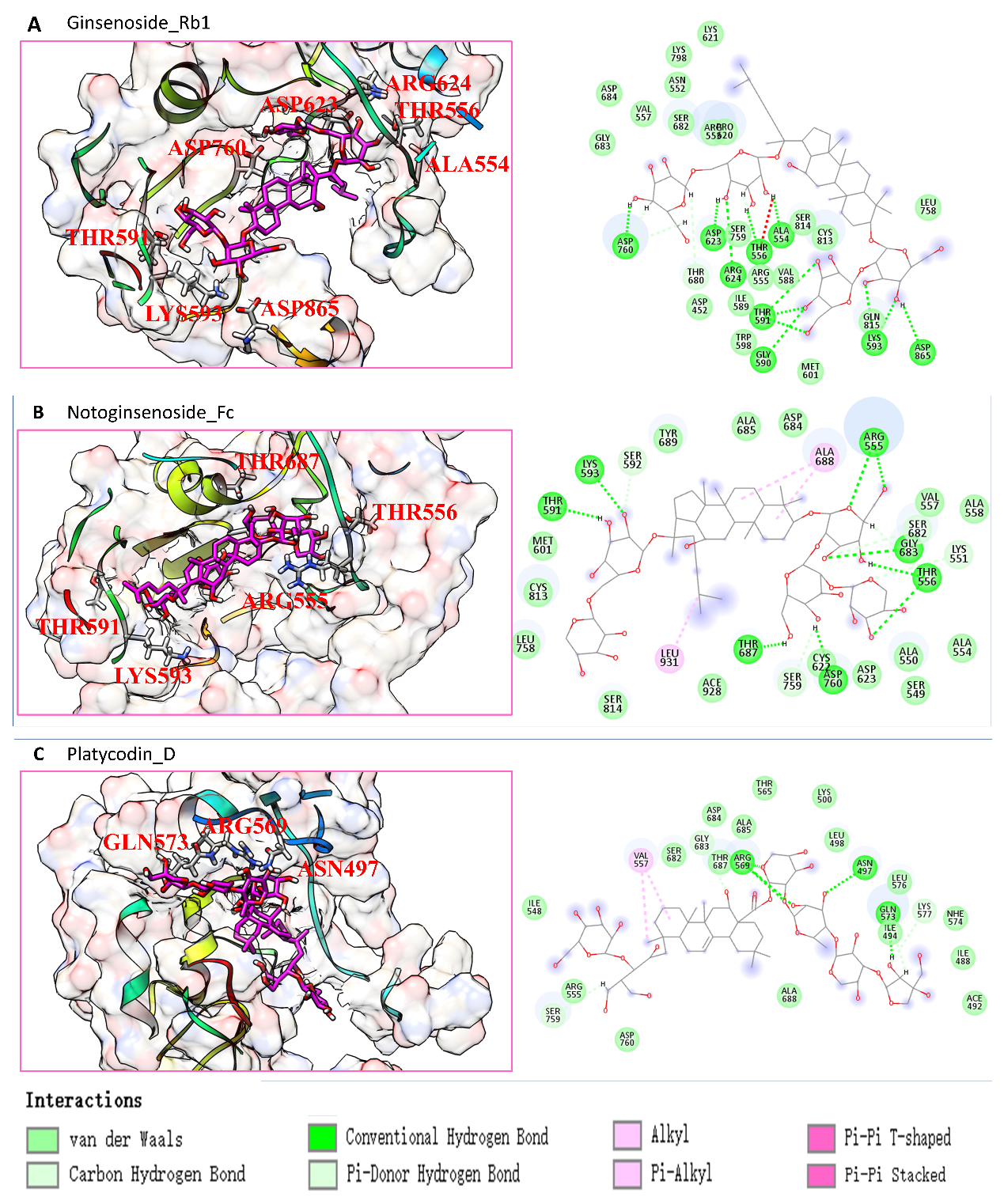


**Figure S2.** The three candidates Ginsenoside_Rb1, Notoginsenoside_Fc, and Platycodin_D interact with the RdRp pocket. A, the last frame of RdRp pocket- Ginsenoside_Rb1 complexes from 100ns MD simulation, as well as its 2D interaction plot; B, the last frame of RdRp pocket-Notoginsenoside_Fc complexes from 100ns MD simulation, as well as its 2D interaction plot; C, the last frame of RdRp pocket-Platycodin_D complexes from 100ns MD simulation, as well as its 2D interaction plot;


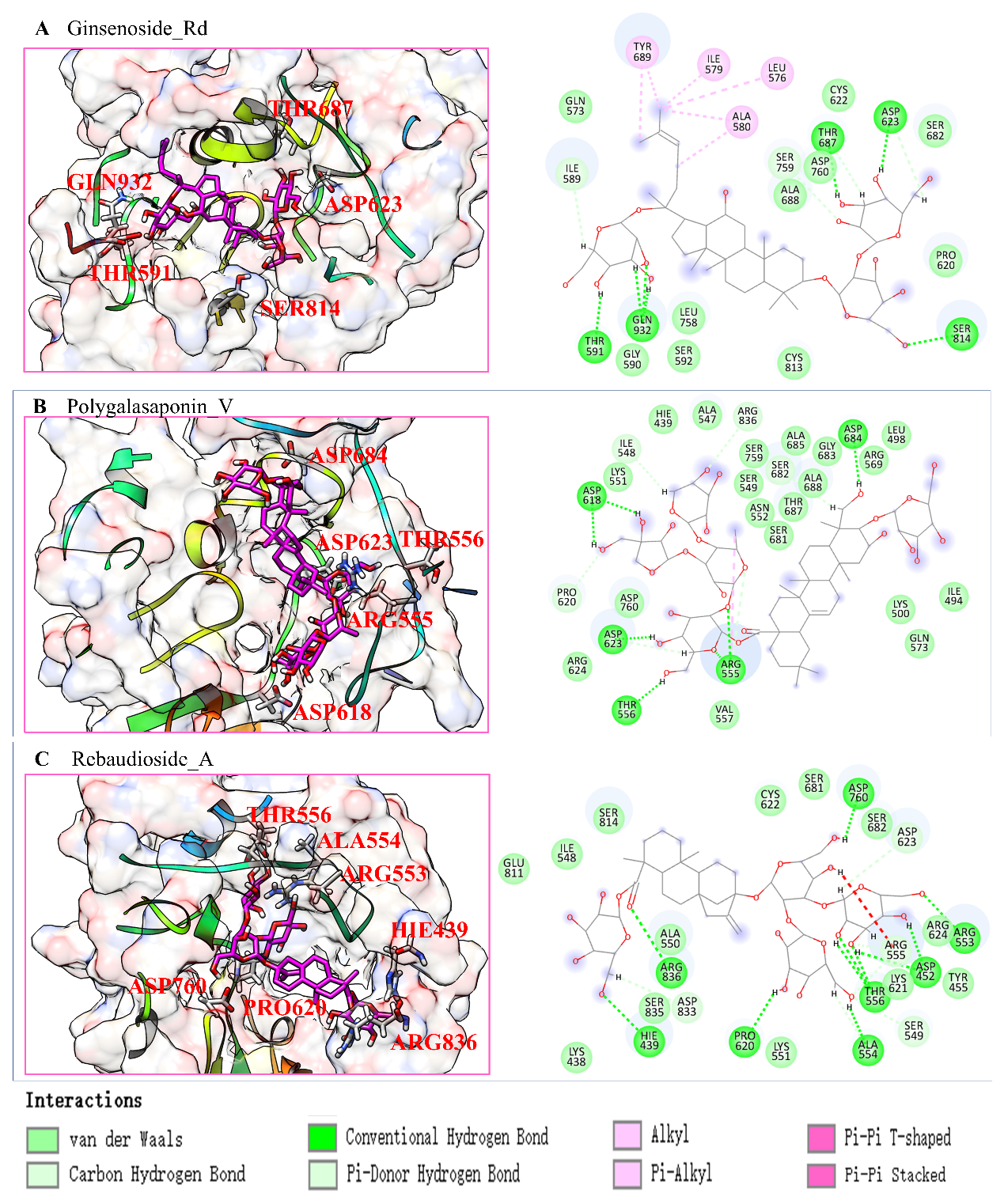


**Figure S3.** The three candidates Ginsenoside_Rd, Polygalasaponin_V, and Rebaudioside_A interact with the RdRp pocket. A, the last frame of RdRp pocket-Ginsenoside_Rd complexes from 100ns MD simulation, as well as its 2D interaction plot; B, the last frame of RdRp pocket-Polygalasaponin_V complexes from 100ns MD simulation, as well as its 2D interaction plot; C, the last frame of RdRp pocket-Rebaudioside_A complexes from 100ns MD simulation, as well as its 2D interaction plot.


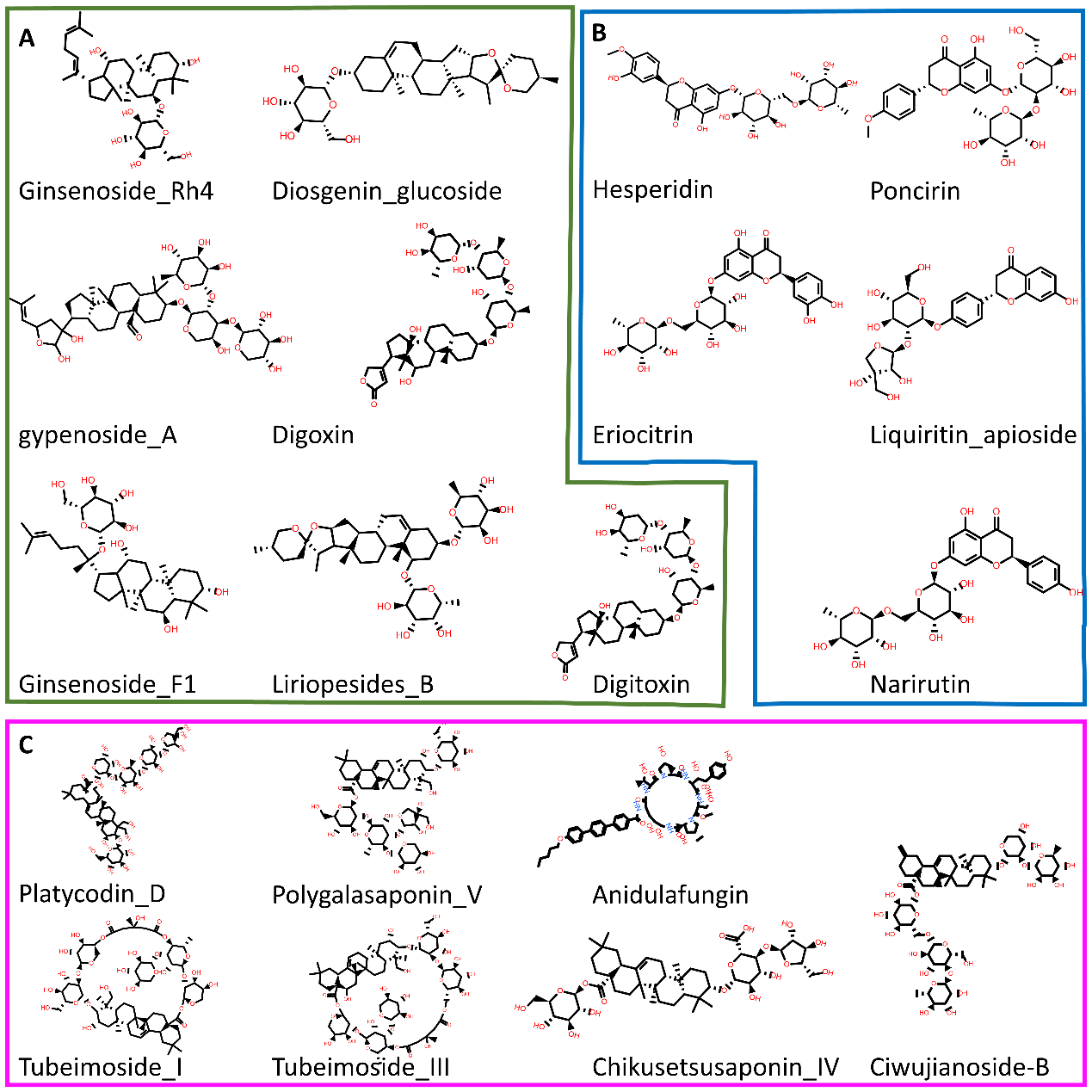


**Figure S4.** The examples of predicted low specificity compounds as well as predicted high specificity compounds. A, the low specificity compounds based on reverse DFCNN; B, the low relative specificity compounds based on reverse docking; C, the high specificity compounds.


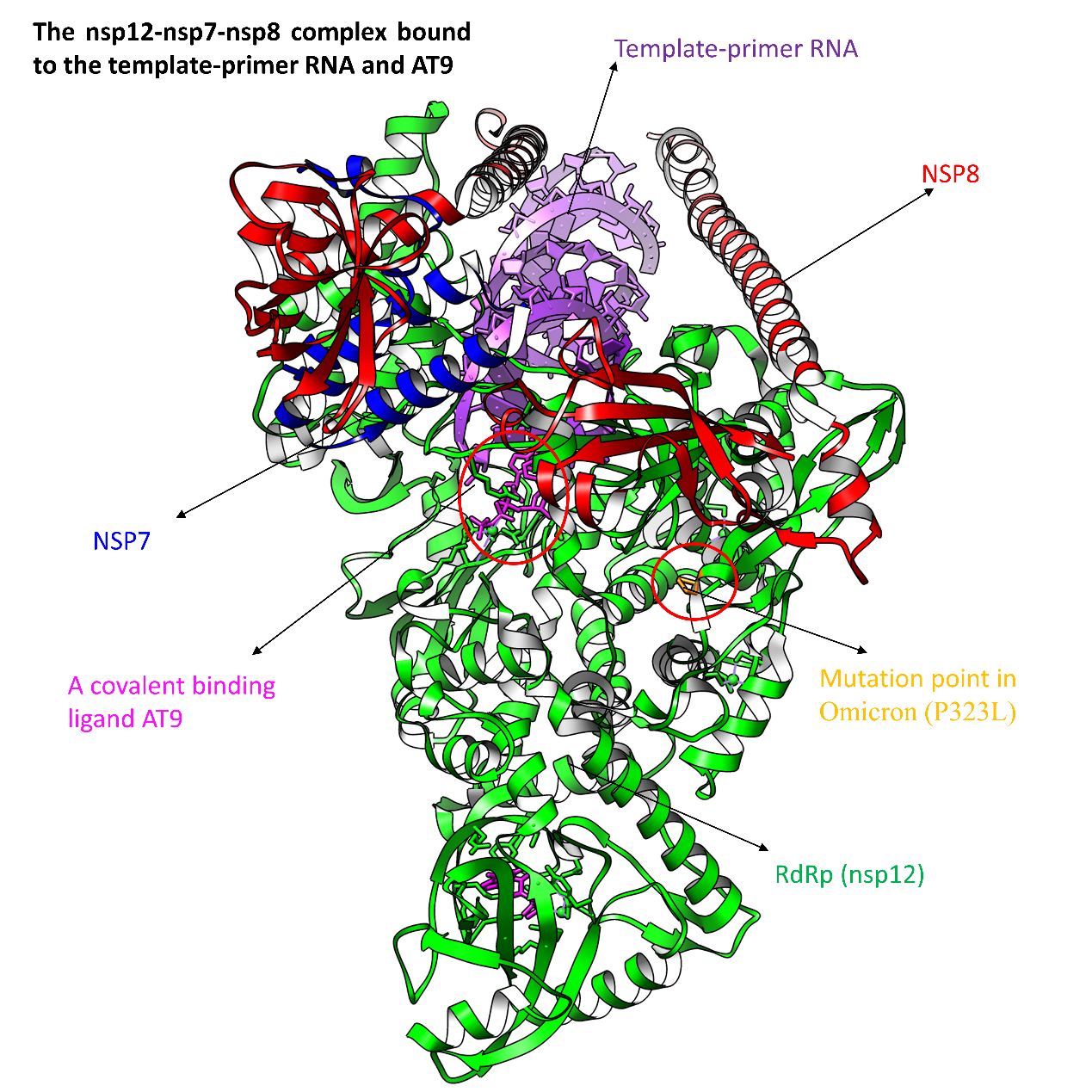


**Figure S5.** Shows nsp12-nsp7-nsp8 complex bound to the template-primer RNA and AT9 with the mutation point in nsp12 of Omicron were marked. The structure was from the PDB with ID 7ED5.
